# A deep learning approach to predict inter-omics interactions in multi-layer networks

**DOI:** 10.1186/s12859-022-04569-2

**Published:** 2022-01-26

**Authors:** Niloofar Borhani, Jafar Ghaisari, Maryam Abedi, Marzieh Kamali, Yousof Gheisari

**Affiliations:** 1 Department of Electrical and Computer Engineering, Isfahan University of Technology, Isfahan, 84156-83111 Iran; 2 Regenerative Medicine Research Center, Isfahan University of Medical Sciences, Isfahan, Iran; 3 Department of Genetics and Molecular Biology, Isfahan University of Medical Sciences, Isfahan, Iran

**Keywords:** Deep learning, Inter-omics interaction prediction, Feature representation, Data Integration

## Abstract

**Background:**

Despite enormous achievements in the production of high-throughput datasets, constructing comprehensive maps of interactions remains a major challenge. Lack of sufficient experimental evidence on interactions is more significant for heterogeneous molecular types. Hence, developing strategies to predict inter-omics connections is essential to construct holistic maps of disease.

**Results:**

Here, as a novel nonlinear deep learning method, Data Integration with Deep Learning (DIDL) was proposed to predict inter-omics interactions. It consisted of an encoder that performs automatic feature extraction for biomolecules according to existing interactions coupled with a predictor that predicts unforeseen interactions. Applicability of DIDL was assessed on different networks, namely drug–target protein, transcription factor-DNA element, and miRNA–mRNA. Also, validity of the novel predictions was evaluated by literature surveys. According to the results, the DIDL outperformed state-of-the-art methods. For all three networks, the areas under the curve and the precision–recall curve exceeded 0.85 and 0.83, respectively.

**Conclusions:**

DIDL offers several advantages like automatic feature extraction from raw data, end-to-end training, and robustness to network sparsity. In addition, reliance solely on existing inter-layer interactions and independence of biochemical features of interacting molecules make this algorithm applicable for a wide variety of networks. DIDL paves the way to understand the underlying mechanisms of complex disorders through constructing integrative networks.

**Supplementary Information:**

The online version contains supplementary material available at 10.1186/s12859-022-04569-2.

## Background

Recent emergence of high throughput technologies has allowed the generation of previously unbelievable amounts of big biological data. The speed of data generation has surpassed data analysis, providing biomedical scientists with tremendous datasets of a size that they have not been encountering before. Hence, big data analysis is a major challenge in modern biology. Although a variety of methods have been developed for omics data analysis in recent years, inter-omics data integration remains a major challenge. It is now commonly believed that the description of biomedical phenomena cannot be reduced to alterations of a single type of biomolecule. Indeed, it is pivotal to consider not only the interactions between one layer of omics data but also complex inter-layer communications to identify the flow of biological information and generate a thorough holistic view of the underlying events.

A number of methods have been developed for omics data integration in order to predict inter-omics interactions. However, they are mostly dependent on specific biochemical properties of network nodes. Hence, their applicability remains restricted to a specific network type. For instance, gene expression data has been used as node features in some previously algorithms [1, 2]. Obviously, these methods can not be applied for the integration of genomics and epigenomics data, for example.

Network embedding, also known as network representation learning, has been recently proposed as a method to embed network nodes into a low-dimensional vector space named latent features, by capturing topological properties of networks and side information. In other words, this method calculates similarity between pairwise nodes to find a low-dimensional manifold structure that is hidden in the corresponding high-dimensional data [3, 4]. One of the methods developed for interaction prediction based on network embedding is matrix factorization, where latent features are detected from network topology [5]. The Data Fusion by Matrix Factorization (DFMF) [6] is a method to predict direct and indirect interactions between heterogeneous nodes. However, these methods are not able to extract highly nonlinear patterns from data. A more recent interaction prediction method, node2vec, learns low-dimensional representations of nodes and tries to maximize the probability of the occurrence of subsequent nodes in random walks over a network. This method has been applied for homogeneous [7] and heterogeneous interaction predictions [8].

Deep learning is a kind of machine learning technique that automatically extracts high-level features from raw data of very large, heterogeneous, high-dimensional datasets. This advantage makes deep learning well suited to the complexity of big data in biology [9–11] as it can be used for network embedding to find complex structural features and learn deep, highly nonlinear node representations [4]. The idea of combining matrix factorization and deep learning is known as deep matrix factorization (DMF). This method extracts representations with two deep neural networks (DNN) and calculates similarity of representations through a cosine function as a non-trainable decoder. DMF is used for recommender systems and has been shown to be superior to traditional matrix factorization [12]. This strategy has recently been used in the prediction of drug–target interactions [13].

Tensor decomposition is a powerful tool for a variety of heterogeneous, sparse, and big data of multi-layer networks [14]. Here, acknowledging the advantages of deep learning and tensor decomposition, an attempt was made to develop an application of deep learning in big biological data integration through employing tensor decomposition by an end-to-end strategy for handling multi-layer networks without relying on specific biochemical features. Data Integration with Deep Learning (DIDL) method is proposed for various kinds of inter-omics interaction prediction. This method consists of an encoder with two DNNs, that extracts representations for biological entities considering node heterogeneity, and a tensor factorization predictor, that predicts the probability of interactions. To demonstrate the applicability of the proposed method, it is evaluated on three different biological datasets, namely drug–target protein, transcription factor (TF)-DNA element, and miRNA–mRNA. Overall, a novel big data integration is proposed that connects heterogeneous layers without being dependent on specific biochemical properties of interacting molecules.

## Methods

Interactions between heterogeneous biomolecules are based on biological principles. For instance, an miRNA targets a group of genes that are functionally related [15, 16], and a TF regulates a bundle of genes that incorporate a specific sequence in their upstream [17]. Hence, the probability of interaction between two given nodes in two different layers can be estimated based on known interactions between each of these two nodes with other elements in the opposite layer. Indeed, unknown interactions can be predicted based on network topology. In this regard, DIDL can serve as an alternative to recommender systems or completion matrix task.

Consider a two-layer network in which two omics layers are linked by inter-omics interactions between heterogeneous biomolecules. If the first and second omics layers contain $n_1$ and $n_2$ biomolecules, respectively, the network structure can be represented by an $n_1 \times n_2$ adjacency matrix $R_{12}$ as follows:

1 $$\begin{aligned} \begin{aligned} R_{12}(i,j) = {\left\{ \begin{array}{ll} 1 \quad \text {if there is interaction between } i_{th} \text { node of the first } \\ \quad \,\, \text {and } j_{th} \text { node of the second omics layer} \\ 0 \quad \text {otherwise} \end{array}\right. } \end{aligned} \end{aligned}$$

where $1\le i \le n_1$, $1\le j \le n_2$ and $R_{12} (i,j)=0$ corresponds to non-interaction or an unknown interaction (an interaction that is not yet investigated). Although both non-interactions and unknown interactions are represented as zero in $R_{12}$, they are different. The DIDL method uses the information of inter-omics interactions to predict new interactions. Remarkably, it is not necessary to know homogeneous interactions in a specific omics layer.

This method has two main components:

- An encoder: two DNNs operating on adjacency matrix and producing latent features for biomolecules of first and second omics layers, and
- A predictor: a tensor factorization model that predict the probability of interactions based on the latent features.

The DIDL method seeks to find the best latent features for representing each biomolecule according to existing interactions. Details of the network structure and model training are given in the following:

### Encoder

As a first stage, encoder extracts the best latent features for representing each biomolecule. For a given biomolecule, its latent feature captures information of all associated kinds of interactions. In this work, two DNNs are proposed to serve as an encoder for extracting high-level features from adjacency matrix $R_{12}$ for inter-omics interaction prediction. As biomolecules of the first and second omics layers are heterogeneous, different DNNs are used for biomolecules of the first and second omics layers. To extract feature vectors of the first layer, the DNN for the first layer, $DNN_1$, takes as input rows of $R_{12}$, which are $n_2 \times 1$ vectors, and produces $k \times 1$ latent feature vectors representing the biomolecules of the first omics layer. Similarly, to extract feature vectors of the second layer while considering heterogeneity of the nodes, the DNN for the second layer, $DNN_2$, takes as input columns of $R_{12}$ which are $n_1 \times 1$ vectors, and produces $k \times 1$ feature vectors representing the second layer nodes. These features have less dimensions than the rows and columns of $R_{12}$, therefore $k<n_1,n_2$.

In order to investigate possible interactions between *i*_th_ biomolecule of the first omics layer and *j*_th_ biomolecule of the second omics layer, (i.e., the pair *i*_th_, *j*_th_), the corresponding latent feature vectors were calculated. The latent feature vector of *i*_th_ biomolecule of the first omics layer and the latent feature vector of *j*_th_ biomolecule of the second one were represented by $U_i$ and $V_j$, respectively. To find $U_i$, the *i*_th_ row of $R_{12}$, which represents inter-omics interactions of *i*_th_ biomolecule of the first layer with biomolecules of the second omics layer, is fed to $DNN_1$ and the output is $U_i$. In a similar way, to find $V_j$,* J*_th_ column of $R_{12}$, which represents inter-omics interactions of *j*_th_ biomolecules of the second omics layer with biomolecules of the first omics layer is fed to *DNN*_2_ and the output is $V_j$. So, the outputs of these DNNs take the following form:

2 $$\begin{aligned} \begin{aligned} U_i&=f_{DNN_1}(R_{12}(i,:))\\ V_j&=f_{DNN_2}(R_{12}(:,j)) \end{aligned} \end{aligned}$$

where $f_{DNN_1}$, $f_{DNN_2}$, $R_{12}(i,:)$ and $R_{12}(:,j)$ are total functions of $DNN_1$ and $DNN_2$ and *i*_th_ and *j*_th_ row and column of $R_{12}$, respectively. Notably, the heterogeneity of biomolecules was herein considered by designing a separate DNN for each omics layer.

### Predictor

Once finished with calculating feature vectors by the encoder, a predictor is devised to apply these feature vectors to investigate existence of interactions. The predictor aims to calculate the probability of interaction between heterogeneous biomolecules. It utilizes latent feature vectors $U_i$ and $V_j$ to assign a score that represents likelihood of interaction between *i*_th_ biomolecule of the first omics layer and *j*_th_ biomolecule of the second one.

In this research, a predictor based on tensor factorization [18] was suggested. On the basis of latent feature vectors $U_i$ and $V_j$, the predictor scores the possibility of interaction through an operation based on tensor factorization, as follows:

3 $$\begin{aligned} \begin{aligned} Score(i,j)=U_i^T D V_j \end{aligned} \end{aligned}$$

in which *Score*(*i*, *j*) measures the probability of interaction between the pair $(i_{th}, j_{th})$ and *D* is a $k \times k$ trainable parameter matrix that models interactions between heterogeneous biomolecules according to the latent feature vectors. As probabilities must logically range between 0 and 1, a sigmoid function was applied on *Score*(*i*, *j*) to calculate the probability of interactions, as follows:

4 $$\begin{aligned} \begin{aligned} \hat{R}_{12}(i,j)=sigmoid(Score(i,j))=\frac{1}{1+e^{-U^T_i D V_j}} \end{aligned} \end{aligned}$$

in which $\hat{R}_{12}(i,j)$ is the probability of interaction between pair (*i*_th_, *j*_th_). In the following, training of the neural network weights and biases of the model for interaction prediction is described.

### Model training and optimization

The encoder maps all biomolecules to latent feature vectors. Then, the predictor predicts probability of interactions. The encoder trains network structure to find the most representative feature vectors for inter-omics interaction prediction. This was done by comparing $R_{12} (i,j)$ predictions $\hat{R}_{12} (i,j)$ against actual data $R_{12} (i,j)$ and calculating an error term that has to be minimized. Thus, DNNs of encoder and tensor factorization predictor are trained by optimizing encoder parameters and matrix *D* using cross-entropy loss, which takes the following form:

5 $$\begin{aligned} \begin{aligned} loss=\frac{1}{m}\sum \limits _{(i,j) \in train \, set} R_{12}(i,j) \log \hat{R}_{12}(i,j)+(1-R_{12}(i,j))\log (1- \hat{R}_{12}(i,j)) \end{aligned} \end{aligned}$$

It adjusts the model to produce high-probability results for interactions (positive samples) and low-probability outcomes for non-interactions (negative samples), with *m* being the number of samples. As encoder and predictor parameters were trained simultaneously, the DIDL became an end-to-end trainable model for inter-omics interaction prediction.

For a multi-layer network, the proposed method needs a list of interactions where each interaction is identified by a triplet (biomolecule in the first omics layer, biomolecule in the second omics layer, interaction identifier). The interaction identifier is 1 (positive sample) if there is interaction between the pair of biomolecules, and 0 (negative sample) if no interaction is between the pair of biomolecules. For proper functioning of the model, the training set in Eq. 5 had to include triplets with positive and negative interactions. The adjacency matrix $R_{12}$ includs information of interactions between heterogeneous biomolecules, with no data on non-interacting biomolecules. Therefore, $R(i,j)=0$ implies an ambiguity between non-interaction or an interaction not yet discoverd. This ambiguity represents a challenge for deep learning methods that rely on both positive and negative interactions for training. In order to solve this challenge, we applied negative sampling. Since negative sampling is equal to 1, therefore the data is balanced. That is, some pairs of biomolecules for which we were unaware of the interaction existence were randomly chosen as negative samples [19]. Actually, every pair of nodes might belong to one of these subsets: positive samples, negative samples, and unknown samples. We do not apply unknown samples in the training, optimizing and evaluating of the model. Positive and negative samples are applied for 10-fold cross-validation. We accept that among the negative samples set, there could be some yet undiscovered interactions. In experimental biology, there is a lack of sufficient data on the absence of interactions. Indeed, only the presence of interactions is commonly shown in wet lab experiments. Hence, negative sampling can be considered as a practical solution to this limitation. This strategy is widely employed in previous studies  [20–22].

According to Eq. 5, the DIDL considers the first-order proximity that means local pairwise proximity between two connected biomolecules [4] across a biological network. In addition, heterogeneous biomolecules exhibiting high second-order proximity share many common neighbors [4], i.e. the rows or columns of the adjacency matrix are similar to each other. Because these rows or columns are the DNNs inputs, biomolecules with high second-order proximity have similar encoder inputs. Consequently, latent features of biomolecules with high second-order proximity become similar, and the DIDL can capture the first-order and second-order proximities simultaneously to preserve the biological network structure.

### Prediction of interaction types

Some omics networks may contain different types of interactions. Consider a network in which two omics layers are linked by *c* types of inter-omics interactions between heterogeneous biomolecules. If the first and second omics layers respectively contain $n_1$ and $n_2$ biomolecules, the network structure can be represented by an $n_1 \times n_2$ adjacency matrix $R_{12}$ as follows:

6 $$\begin{aligned} \begin{aligned} R_{12}(i,j) = {\left\{ \begin{array}{ll} 1 \quad \text {if there is } 1_{th} \text { kind of interaction between pair } (i,j) \\ \vdots \quad \\ c \quad \text {if there is } c_{th} \text { kind of interaction between pair } (i,j) \\ 0 \quad \text {otherwise} \end{array}\right. } \end{aligned} \end{aligned}$$

The encoder is the same as the binary model. In predictor, for $t_{th}$ type of interaction that $1\le t \le c$, there exists the matrix of $D_t$. The matrix $D_0$ is also considered for the absence of interaction. The scores of each kind of interaction is obtained through tensor factorization operation:

7 $$\begin{aligned} \begin{aligned} s_0&=U^T_i D_0 V_j\\ s_1&=U_i^T D_1 V_j \\&\vdots \\ s_c&=U_i^T D_c V_j \end{aligned} \end{aligned}$$

Values of $s_t$ are scores. Then, these scores are passed through a softmax layer. The equation for the softmax function is as follows:

8 $$\begin{aligned} \begin{aligned} softmax(s_t)=\frac{e^{s_t}}{\sum \limits _{l=0}^c e^{s_l}} \end{aligned} \end{aligned}$$

that:

9 $$\begin{aligned} \begin{aligned} softmax(\overrightarrow{S})= \begin{bmatrix} P(\hat{R}_{12}(i,j)=0) \\ P(\hat{R}_{12}(i,j)=1)\\ \vdots \\ P(\hat{R}_{12}(i,j)=c)\\ \end{bmatrix} \end{aligned} \end{aligned}$$

Each value in the output of the softmax function can be interpreted as the probability of each type of interaction or no interaction. Furthermore, the loss function was changed to categorical cross-entropy. The advantage of this method is that not only predicts the existence of interaction but also the types of interactions.

### Experimental setup

The DIDL method is implemented based on Tensorflow. The encoder was developed with pairs of 4-layer neural network architectures with the Relu activation functions and two 64 and 32 hidden units in the first and second hidden layers, respectively. The latent feature vector dimension, *k*, was set to 20 for all three datasets and the batch sizes were 32, 32, and 1024 for the miRNA–mRNA dataset, the drug–target dataset, and the TF–DNA dataset, respectively. The model parameters were randomly initialized with a Gaussian distribution with zero mean and a standard deviation of 0.01. To optimize the model, the Adam optimizer [23] was utilized with a learning rate of 0.0001. In order to improve the generalization of the model for the prediction of unforeseen inter-omics interactions, the drop out and *L2* normalization for encoder weights and *D* matrix were applied and set to 0.5 and 0.08, respectively. The random search was further applied for hyperparameter tuning. In kind of interaction prediction, the encoder’s DNNs had 512 and 128 hidden units in the first and second hidden layers, respectively. The latent feature vector dimension, *k*, was set to 64.

Performance of the developed DIDL was evaluated against the node2vec, DeepWalk [24], Common Neighbor (CN), and Jaccard Index (JI) [25] methods. In node2vec and DeepWalk methods, the window size, walk length, walks per vertex and dimensions were set to 10, 40, 10, and 20, respectively.

## Results

**Fig. 1:**
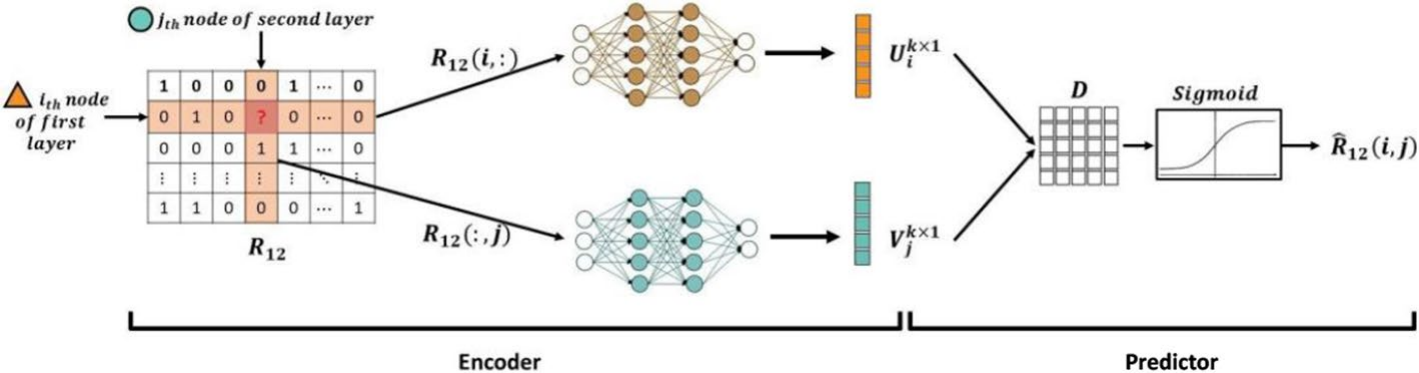
Overview of DIDL method framework. Rows and columns of $R_{12}$ are fed to an encoder which consists of two DNNs. The outputs of the encoder are latent features of the biomolecules. Finally, these latent features are transformed into a tensor factorization predictor and its output indicates the probability of interaction between heterogeneous biomolecules

Recently, tremendous generation of omics data provides a unique opportunity to construct holistic maps for complex disorders. However, construction of integrative networks is limited due to lack of sufficient data about the interactions between heterogeneous biological entities, a problem that has come to some sort of solution by the emergence of machine learning methods. In this study, DIDL was developed as a deep learning-based method for big biological data integration, where “encoder” extracts representation vectors based on existing interactions and negative samples, followed by predicting the probability of interactions by “predictor” (Fig. 1). To assess the performance of the developed method, it was applied on three different heterogeneous biological datasets: drug–target protein, TF–DNA element, and miRNA–mRNA. In addition, to assess the performance of DIDL in a more complicated situation, it is applied on the Hetionet [26] network which includes interactions between 11 different layers.

### Drug–target interaction prediction

**Fig. 2:**
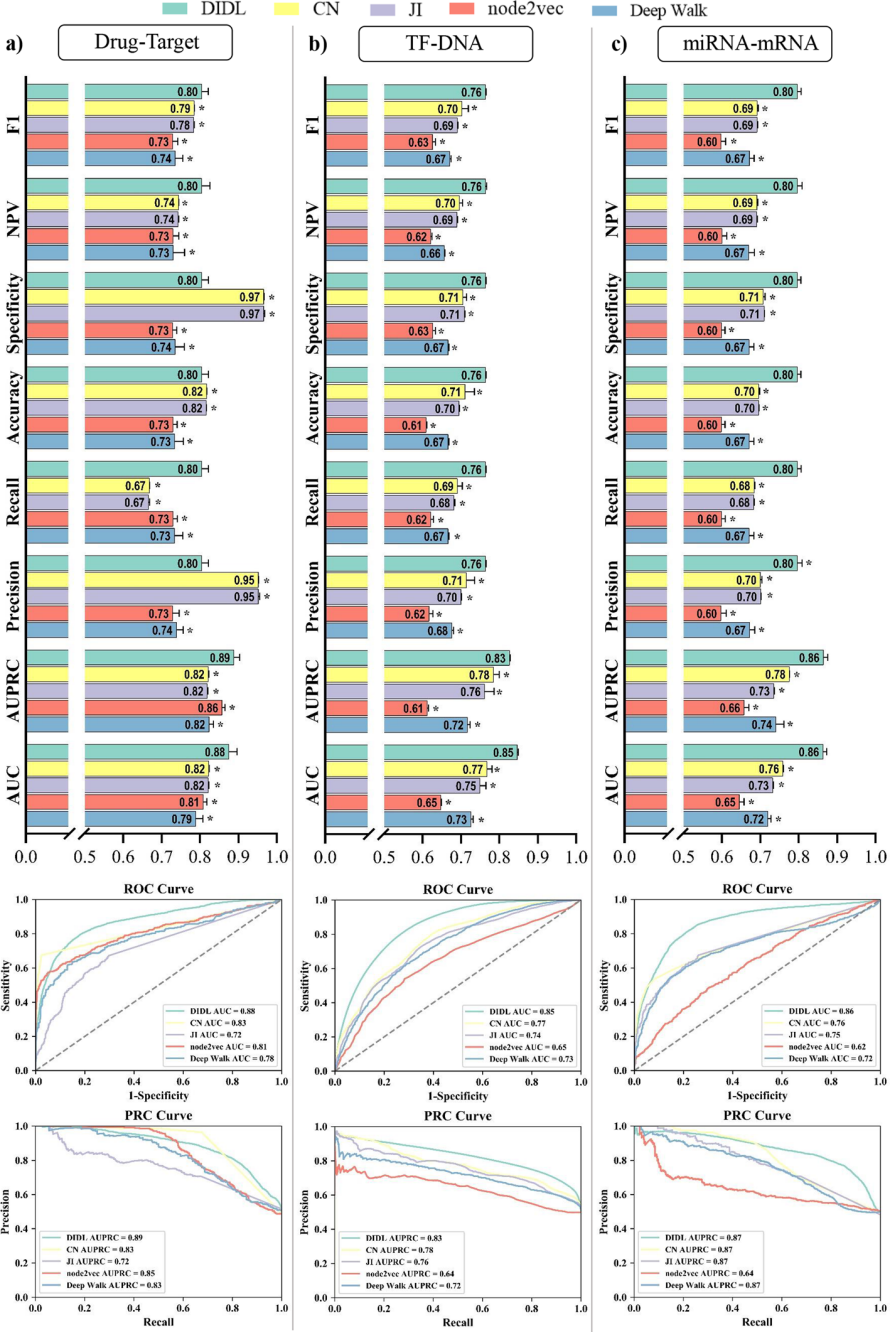
Evaluation of DIDL using 10 fold cross-validation. The performance of DIDL for interaction prediction in drug–target (**a**), TF–DNA (**b**), and miRNA–mRNA (**c**) is assessed using different measures and compared with node2vec, DeepWalk, CN, and JI

Drug repositioning or repurposing is a promising approach in drug discovery. In recent years, a few strategies have been developed for drug repurposing, which are known to suffer from particular disadvantages, including their need for retrieving huge amounts of biological information from the literature or existing databases [27]. To demonstrate the capability of the proposed model in this research for drug repositioning, the DIDL method was employed to predict new links between drugs and proteins. For this purpose, known drug–target interactions were extracted from DrugBank database [28]. This dataset covers a total of 1507 drugs, 1642 target proteins, and 6439 interactions (Additional file 1). To evaluate the application of DIDL for drug target prediction, a 10-fold cross-validation procedure was performed and different indices were evaluated, including area under receiver operating characteristic curve (AUC), area under precision–recall curve (AUPRC), precision, recall, and accuracy measures [29]. DIDL was further assessed using comparison with node2vec [7], DeepWalk [24], CN, and JI [25]. DIDL could outperform the mentioned methods, as per the outcomes of *T *test analysis (*P* value < 0.05, Fig. 2a).

To further evaluate the performance of DIDL, it is compared with the GCN-DTI method, which in addition to known interactions between drugs and proteins, utilizes drug-drug interactions and protein–protein interactions data [20]. Although DIDL method does not use such information, the difference in performance of these two methods is subtle. Indeed, in spite of using less information, the output is in the same order (Additional file 2). Noteworthy, the reliance of DIDL only on inter-layer interactions, not intra-layer data, allows its application for a wide spectrum of networks.

### TF–DNA interaction prediction

Transcriptional regulation of gene expression is a result of the interactions of TFs with specific DNA sequence elements named transcription factor binding sites (TFBSs), which is a critical step to control cell behaviors. The existing knowledge about these interactions is preliminary. Current algorithms use data derived from chromatin immunoprecipitation followed by microarray (ChIP-chip) or sequencing (ChIP-Seq) techniques or rather apply a combination of in-silico sequence motif detection with experimental data for prediction of TF–TFBS interactions. However, their performances are limited due to insufficient data [30]. To assess validity of DIDL for predicting the link between TFs and TFBSs, known experimental data on human TF–TFBSs were extracted from the Enrichr database using ChEA 2016 [31]. This dataset contains data on a total of 175 TFs, 35116 genes, and 407245 interactions (Additional file 3). This data on known TF–DNA interactions was exploited by DIDL to predict unforeseen interactions. A 10-fold cross-validation scheme was used and performance indices were also measured. Once more, node2vec, DeepWalk, CN, and JI were also applied and performances were evaluated in terms of AUC, AUPRC, precision, recall, and accuracy measures. Notably, DIDL outperformed the mentioned methods, as indicated by all of the indices (*P* value $<0.05$, Fig. 2b).

### miRNA-mRNA interaction prediction

The complexity of the RNA world has been increasingly appreciated in recent decades. The miRNA is a key regulator of a variety of cellular processes and identification or prediction of its interaction with mRNA is yet a major challenge. Despite huge efforts, current tools still have suboptimal performance and even the best available algorithms have low accuracy and sensitivity [30, 32]. In order to assess the performance of DIDL in miRNA target prediction, experimentally validated human miRNA–mRNA interactions were retrieved from miRTarBase 7.0, and a total of 8112 interactions with strong evidence for 735 miRNAs and 2746 mRNAs were chosen (Additional file 4). Next, DIDL was employed to predict further interactions, ending up with good performance, as per indices equaling or exceeding 0.8. Remarkably, DIDL method also outperformed node2vec, DeepWalk, CN, and JI, as revealed by the results of *T *test analysis (*P* value $< 0.05$, Fig. 2c).

**Fig. 3:**
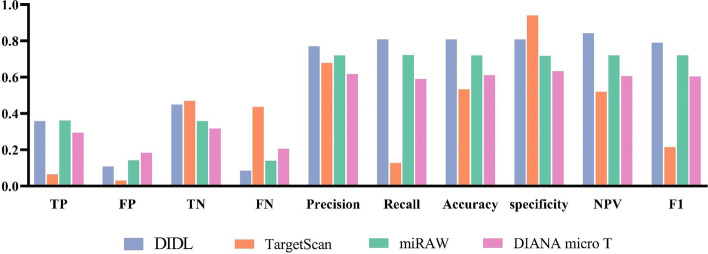
The validity of DIDL for miRNA–mRNA prediction The performance of DIDL for prediction of miRNA targets is compared with TargetScan, miRAW, and DIANA microT. The exploited dataset is harvested from the study of Pla et al. 11

The proposed method was further compared with some state-of-the-art methods. The miRAW dataset was harvested from the study by Pla et al. [11] to compare DIDL with TargetScan (conserved) [33], miRAW (7–2:10 AE) [11] and DIANA microT [34]. This dataset consists of 449 miRNA, 6318 mRNA, 33142 positive samples, and 32248 negative samples. In this dataset, non-functional interactions have been assumed as negative samples. Comparing DIDL method with the existing algorithms, it was figured out that this proposed method was superior to other state-of-the-art target prediction methods (Fig. 3). The measures of TargetScan, miRAW, and DIANA microT for miRAW data set are harvested from Pla et al. study [11].

Although the validity of DIDL was evaluated per various indices, we further evaluated the method by performing a literature survey for novel interactions. The probability of interaction between every heterogeneous pair of miRNA and mRNA was determined using the developed algorithm and the interactions were sorted based on their probability scores. Interestingly, 7 out of 10 top predicted interactions were confirmed by experimental investigations that are not yet incorporated in miRTarBase (Table 1). These validation strategies underscore the applicability of DIDL for miRNA target prediction.

DIDL encoder cluster omics elements through latent feature extraction. According to Eq. 3, matrix *D* with the dimension of $k\times k$ encodes association between latent features. That is, *D* is a low dimensional compression of $R_{12}$. Therefore, the unknown interactions are inferred based on the cluster association.

**Table 1:** Novel miRNA target predictions with the highest probability scores by the DIDL

Rank	miRNA	mRNA	Probability	Evidence
1	hsa-miR-15b-5p	ZEB1	0.9974	[41]
2	hsa-miR-34c-5p	EZH2	0.9973	[42]
3	hsa-miR-15b-5p	EZH2	0.9973	[43]
4	hsa-miR-34c-5p	ZEB1	0.9973	[44]
5	hsa-miR-34c-5p	TGFBR2	0.9972	*hsa-miR-34a, 34b [45]
6	hsa-miR-30c-5p	EZH2	0.9972	*hsa-miR-30d [46]
7	hsa-miR-15b-5p	RUNX2	0.9972	[47]
8	hsa-miR-15b-5p	TGFBR2	0.9971	[48]
9	hsa-miR-183-5p	SIRT1	0.9971	[49]
10	hsa-miR-34c-5p	FOXO1	0.9971	*hsa-mirR-34a, 34b [50]

The first column indicates the rank of predicted miRNA-mRNA pairs based on the probability score. Experimental supports for the predictions are cited in the last column. Asterisks denote that the experimental evidence is for another member of the same miRNA family

**Fig. 4:**
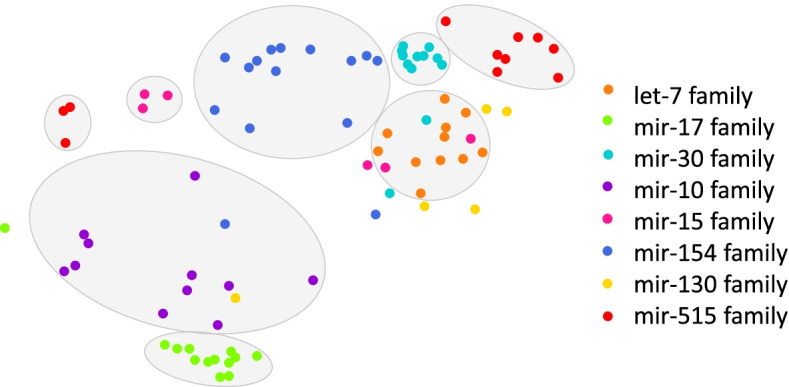
T-SNE Visualization of miRNA latent features. T-SNE analysis on latent space of DIDL for miRNA–mRNA interaction prediction indicates that miRNAs are clustered according to their families. Families with less than four members are not shown

We employ the T-SNE (T-distributed stochastic neighbor embedding) algorithm to visualize the node feature vectors learned by the encoder. The T-SNE is a non-linear dimensionality reduction strategy that embeds similar objects in high-dimensional space close to each other in a reduced dimension space [35]. Using T-SNE, the latent features of miRNAs are projected to a two-dimensional space. Although DIDL was not provided with any biological features of miRNAs, the T-SNE analysis demonstrated that miRNAs were clustered according to their families (Fig. 4). This observation can be explained by the fact that in miRNA–mRNA networks, miRNAs which are in the same family have similar seed sequences and hence similar targets. Indeed, DIDL clusters miRNAs based on their interactions in an unsupervised manner and it is in concordance with miRNA families. This is strong evidence for the validity of this algorithm.

### Prediction of interaction in a complex multi-layer network

To assess the performance of DIDL for interaction prediction in a more sophisticated context, it is applied on the multi-layer Hetionet network [26]. DIDL was exploited for every two layers and could successfully predict interactions in such a complex network (Additional file 5).

### Prediction of interaction types

**Fig. 5:**
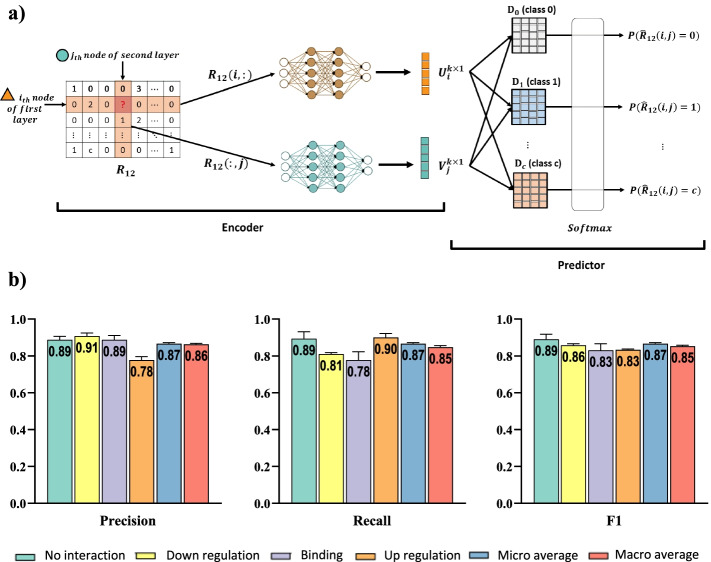
Overview of modified DIDL for kind of interaction prediction. (**a**) In order to predict the kind of interactions, the prediction is extended by having $c+1$ matrix *D* that *c* is the number of kinds of interactions. (**b**) The performance of the algorithm for the prediction of three different kinds of interaction between small molecule compounds and genes in the Hetionet network is demonstrated

To predict the types of interactions, we are faced with a classification problem with the $c+1$ class that *c* is the number of types of interactions. In addition, the elements of the adjacency matrix become the class number of interaction types, and the predictor is extended by having a *D* matrix for every type of interaction and $D_0$ for no interaction (Fig. 5a).

A modified version of DIDL that can predict the types of interactions was developed and its validity was assessed using a part of the Hetionet dataset [26]. We used the Gene-Compound layers in this dataset consisting of three different types of interactions: upregulation, downregulation, and binding. DIDL could successfully not only predict the presence of interactions but also their types (Fig. 5b)

### Robustness of DIDL to network sparsity

**Fig. 6:**
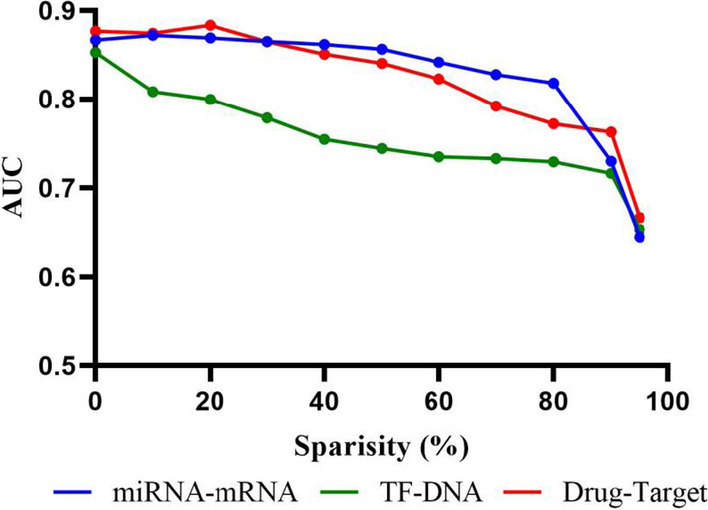
Impact of network sparsity. The performance of DIDL is robust to network sparsity

Big biomedical data is often highly dimensional but sparse [36]. As the presented method is based on adjacency matrix of the biological networks, sparsity of the adjacency matrix is potentially an important factor in the modeling performance. Hence, to assess robustness of the method to network sparsity, 10% of the interactions were held out as the testing subset and then the sparsity of the remaining network was gradually increased by random removal of a portion of the remaining interactions in the training set. As expected, by increasing network sparsity, the model performance degraded. However, the model performance remained acceptable until removing around 50% of interactions, especially for miRNA–mRNA and drug–target datasets (Fig. 6).

### Assessment of the effect of encoder

To investigate the real impact of the encoder, it is removed and rows and columns of the adjacency matrix of interactions are fed directly to the tensor factorization predictor. This modification makes the model functionality absolutely reduced (Additional file 6). Especially for miRNA-mRNA and drug–target datasets, it becomes near to random. This experiment underscores the importance of encoder in the proper performance of the model.

## Discussion

In order to achieve holistic views towards the complex mechanisms of physiological or pathological phenomena, it is imperative to construct multi-layer networks that consider interactions of heterogeneous biomolecules. This study was aimed at developing a highly nonlinear mathematical data integration method based on deep learning for interaction prediction between any two layers of biological networks on the basis of known interactions. The encoder and predictor were simultaneously trained according to rows and columns of adjacency matrix of network interactions. DIDL efficiency was assessed for interaction prediction on drug–target, TF–DNA, and miRNA–mRNA networks and compared with alternative methods. Also, the validity of predictions was assessed by literature surveys. Furthermore, an enhanced version of DIDL was developed which can predict the kind of interactions.

We appreciate that DIDL is a combination of multilayer perceptron (MLP) and tensor factorization, but this combination works more effectively and is applicable for link prediction in different kinds of multi-omics heterogeneous networks without dependence on biological properties of interacting elements. Additionally, some available methods for the prediction of interactions between two heterogeneous layers rely on homogenous interactions inside each layer. DIDL overcomes this limitation as it is trained solely with known inter-layer interactions. These advantages make the developed algorithm a suitable choice specially for cases such as miRNAs that neither the intra-layer interactions nor the biological features of nodes and mechanisms of interactions are comprehensively discovered. DIDL was found to outperform even the best available algorithms for miRNA–mRNA interaction predictions, such as TargetScan, miRAW, and DIANA microT. Notably, the visualizing of latent features with T-SNE showed that although DIDL was not provided with biological information of miRNAs, it could cluster them based on their families. This is strong evidence for the validity of this algorithm.

Large-scale investigations on interactions between biomolecules including proteins have just recently begun and a majority of interactions are possibly yet undiscovered. Hence, considering the dependency of DIDL to recognized interactions, we were interested to know how robust this method is to network sparsity. We observed that DIDL retains an acceptable level of performance after removing a considerable fraction of known interactions in the training subset. This suggests that even in the current situation where molecular connections are not completely understood, DIDL can be reliably exploited.

Another advantage of the proposed method is that the processes of feature selection and network representation are automatic. Although the logic of the method for predicting new interactions is based on the previous interactions, the tendency of nodes toward interaction can vary depending on the network type. For example, in a PPI network, the probability of interaction between two proteins sharing many common neighbors is actually low [37]. On the contrary, in a gene-disease network, genes causing the same or similar diseases tend to interact with one another  [38]. Therefore, manual feature extraction is not a good choice especially when the network behavior is not properly known.

DIDL is a novel autoencoder architecture that is capable of learning a joint representation of both first-order and second-order proximities. This architecture provides for efficient end-to-end training in a single learning stage to simultaneously perform node representation and link prediction. In this way, the predictor and encoder parameters can be jointly optimized. Recent research indicates that the modeling of graph-structured data can be considerably enhanced with such an end-to-end learning scheme [39, 40]. This can, at least partly, describe the superiority of the DIDL over node2vec, DeepWalk, CN, and JI.

In conclusion, using a deep learning strategy, we have here proposed a novel inter-omics prediction pipeline that relies on minimum data and is applicable for various kinds of networks. It can be exploited to construct multi-layer networks and generate comprehensive maps of the underlying mechanisms of complex disorders.

## Supplementary information

**Additional file 1:** Interaction list for drug–target. The raw data used in the drug–target is included in this file. Drug–target interactions were extracted from DrugBank database

**Additional file 2:** Comparison between DIDL and GCN-DTI method. The DIDL is compared with GCN-DTI method in Yamanashi dataset. The measures of GCN-DTI are harvested from Zhao et al. [20]

**Additional file 3:** Interaction list for TF–DNA. The raw data used in the TF–DNA is included in this file. Interactions were extracted from the Enrichr database using ChEA 2016

**Additional file 4:** Interaction list for miRNA-mRNA. The raw data used in the miRNA-mRNA is included in this file. miRNA-mRNA interactions were collected using miRTarBase 7.0

**Additional file 5:** Evaluation of DIDL with Hetionet network. Performance of DIDL for prediction of interactions between different layers of Hetionet network is assessed using 10 fold cross-validation

**Additional file 6:** Impact of the encoder. Removing the encoder significantly declines the performance of DIDL

## Data Availability

Not applicable.

## Abbreviations

DFMF: Data fusion by matrix factorization
DMF: Deep matrix factorization
DNN: Deep neural networks
DIDL: Data integration with deep learning
TF: Transcription factor
AUC: Area under receiver operating characteristic curve
AUPRC: Area under precision–recall curve
CN: Common neighbors
JI: Jaccard Index
